# Acupuncture Decreases Blood Pressure Related to Hypothalamus Functional Connectivity with Frontal Lobe, Cerebellum, and Insula: A Study of Instantaneous and Short-Term Acupuncture Treatment in Essential Hypertension

**DOI:** 10.1155/2016/6908710

**Published:** 2016-09-04

**Authors:** Yu Zheng, Jiping Zhang, Yanjie Wang, Yuying Wang, Yujun Lan, Shanshan Qu, Chunzhi Tang, Yong Huang

**Affiliations:** ^1^School of Traditional Chinese Medicine, Southern Medical University, Guangzhou, Guangdong Province 510515, China; ^2^Clinical School of Acupuncture and Rehabilitation, Guangzhou University of Chinese Medicine, Guangzhou, Guangdong Province 510405, China

## Abstract

The therapeutic effects of acupuncture in decreasing blood pressure are ambiguous and underlying acupuncture in hypertension treatment has not been investigated. Our objective was to observe the change of quality of life and compare the differences in brain functional connectivity by investigating instantaneous and short-term acupuncture treatment in essential hypertension patients. A total of 30 patients were randomly divided into the LR3 group and sham acupoint group. Subjects received resting-state fMRI among preacupuncture, postinstantaneous, and short-term acupuncture treatment in two groups. Hypothalamus was selected as the seed point to analyze the changes in connectivity. We found three kinds of results: (1) There was statistical difference in systolic blood pressure in LR3 group after the short-term treatment and before acupuncture. (2) Compared with sham acupoint, acupuncture at LR3 instantaneous effects in the functional connectivity with seed points was more concentrated in the frontal lobe. (3) Compared with instantaneous effects, acupuncture LR3 short-term effects in the functional connectivity with seed points had more regions in frontal lobe, cerebellum, and insula. These brain areas constituted a neural network structure with specific functions that could explain the mechanism of therapy in hypertension patients by LR3 acupoint.

## 1. Introduction 

Essential hypertension (EH) is a common chronic disease, which affects up to one billion individuals worldwide and is attributable each year to more than 7 million deaths and loss of 64 million disability-adjusted life years [1]. The Global Burden of Disease study estimates 62% of stroke, 49% of ischemic heart disease, and 14% of other cardiovascular disease can be attributable to mean systolic blood pressure levels >115 mmHg worldwide [2]. Blood pressure can be lowered by several classes of drugs and by such lifestyle changes as weight loss, salt intake restriction, and exercises [3]. However, due to various side effects or safety concerns, such as drug resistance which could affect therapeutic efficacy, antihypertensive medication is unsatisfactory. In addition, lifestyle interventions are difficult to achieve and even more difficult to maintain.

Acupuncture, one of the oldest and most commonly used forms of alternative medicine, has existed for 2500 years [4], which has been reported to have potential for treating cardiovascular diseases, including EH [5–9]. However, these therapeutic effects of acupuncture in lowering BP are ambiguous. Meanwhile, these studies have only observed the efficacy of it; the mechanism underlying acupuncture in high BP treatment has not been investigated.

In the past decades, fMRI studies [10–12] have demonstrated that acupuncture stimulation can modulate brain activities. Some cerebrovascular disorders have been linked to reduce brain network functioning that include the default mode network (DMN), which represents a resting-state network that consists of a set of brain regions that coactivate when subjects are at rest and deactivate together when subjects become engaged in external cognitive tasks [13]. But the cardiovascular center is mainly located in the medulla oblongata and hypothalamus, and these areas are not in the DMN [14]. Many studies [15–17] had shown that the hypothalamus may be the key role in the central nervous system (CNS) in cardiovascular regulation, especially the supraoptic (SO) nucleus and paraventricular (Pa) nucleus of the hypothalamus, which can produce the vasopressin.

The study of quality of life is an important part in chronic disease [18]; many researchers paid attention to hypertension patients [19–21]. But most of the questionnaires in their studies were SF-36, which could not reflect the most common and most direct questions affecting the quality of life in patients with hypertension.

Our present study attempted to investigate instantaneous and short-term acupuncture treatment in EH patients using resting-state fMRI, SF-36, and QLICD-HY [22] questionnaires. We aimed to perform a clinical trial in patients with hypertension to evaluate the change of quality of life and compare the differences in brain functional connectivity between preacupuncture and postinstantaneous and short-term acupuncture treatment. We hope to reveal the possible mechanism of acupuncture for treating hypertension in the hypothalamus related brain network.

## 2. Methods

### 2.1. Participants

100 hypertension participants were recruited from different community hospitals in Guangzhou. The inclusion criteria were as follows: (1) mild or moderate arterial hypertension, grade 1 or 2 of the European Society of Hypertension-European Society of Cardiology Guidelines 2003 [23], with the use of antihypertensive drugs but with a clear history of hypertension, with species and dose of antihypertensive medicine not being changed during the study period; (2) age between 45 and 65 years and right handedness; (3) regular diet with minimal liquor, tobacco, tea, and coffee consumption and normal sleeping patterns (before 12 a.m.); moderate-sized body mass index of 18.5–23.9 (Chinese); no history of nervous system disease; (4) no pain (including dysmenorrhea) or insomnia within 1 month before the test; (5) no metallic substances in the body, such as stents; (6) no noise exposure and hypothermia; no fear of confined spaces; (7) no acupuncture procedure within 1 month before the test. The drop-off criteria were as follows: (1) hypertensive crisis or other emergency; (2) inability to follow this study because of personal reasons. The subjects were informed about the experiment and voluntarily signed informed consent in advance. Only 30 participants passed the screening. This experiment was approved by the Chinese Ethics Review Committee (ChiECRCT-2012011) and was registered at the Chinese Clinical Trial Registry (ChiCTR-TRC-12002427).

According to the complete randomized block design, the 30 subjects were distributed into two groups, with each group consisting of 15 people. There were no statistically significant differences between the groups in terms of baseline characteristics (*P* > 0.05; Table 1).

### 2.2. Trials and Processing Methods

**Figure 1:**
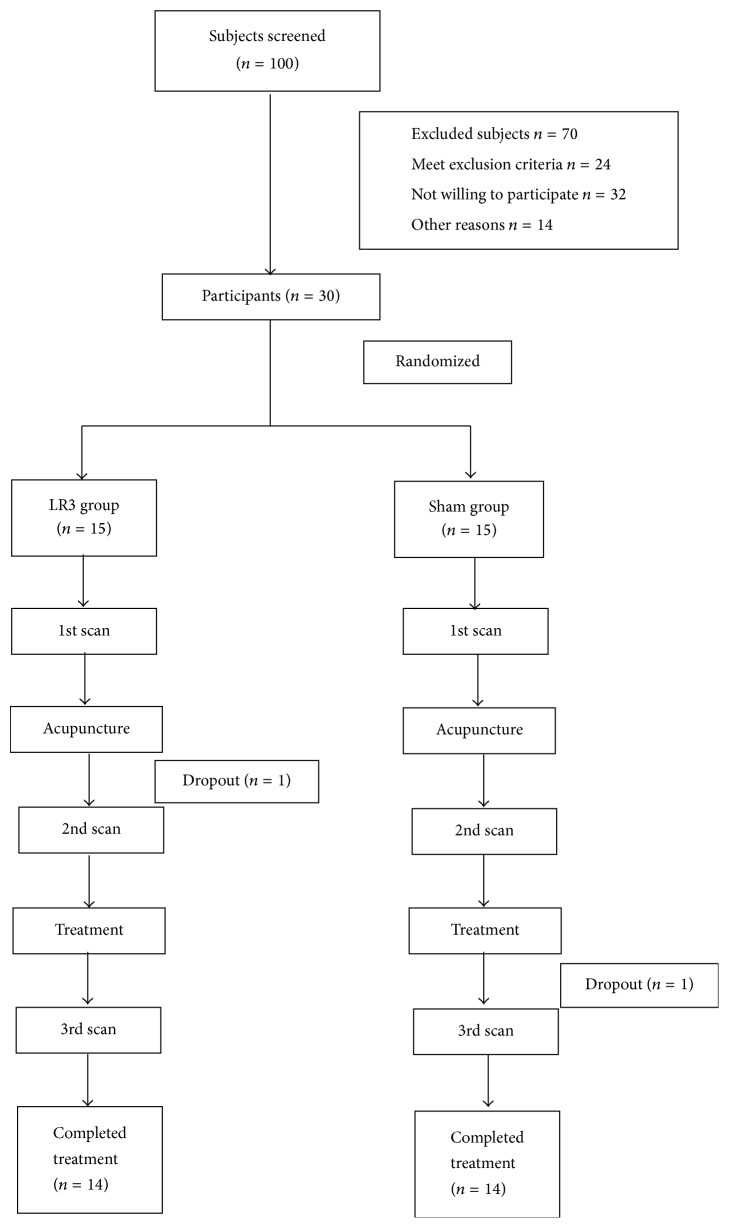
Flow chart.

The LR3 group subjects underwent LR3 acupoint. The other group subjects underwent sham point. The blood pressure (BP) of the participants was measured before and after MRI scan. The BP measurements are performed with mercurial sphygmomanometer (Jiangsu Yuyue Medical Equipment & Supply Co., Ltd.). Considering that BP can be easily affected by psychological factors, participants filled out questionnaires for assessments of anxiety (State Trait Anxiety Inventory, STAI). After a first acupuncture session of 30 minutes, defined to be a “once acupuncture” session, all participants underwent 10 acupuncture treatments in two weeks (once a day except weekends), with each session lasting about 30 minutes. This group's subjects were asked to complete SF-36 and QLICD-HY questionnaires before and after the treatment. Subjects were asked to pass urine and stool prior to treatment. The patients' eyes were masked with eyeshades, and earplugs were simultaneously worn so that their audiovisual system could not be stimulated. The flow chart is as follows (Figure 1).

#### 2.2.1. Acupuncture Interventions

**Figure 2:**
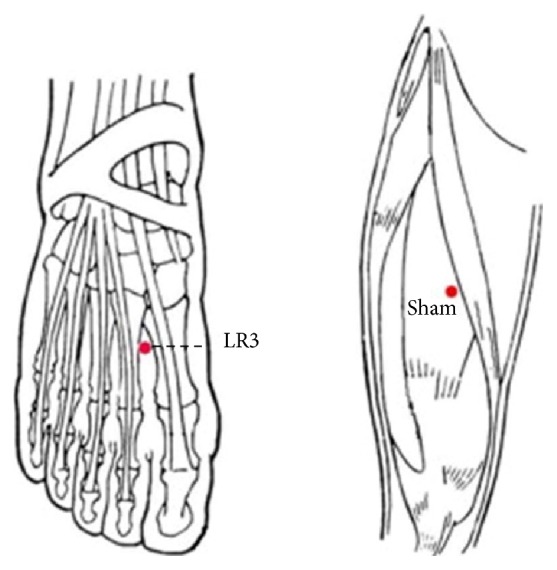
The location of the acupoints.

LR3: it is on the dorsum of the foot, in the depression anterior to the junction of the first and second metatarsal bones (Chinese National Standards GB/T12346) [24] (Figure 2).

Sham point: it is on the midpoint of the line connecting the anterior superior iliac spine and lateral border of the patella, 2 cm inside.

After local skin disinfection and sterilizing with alcohol, sterile acupuncture needles (0.3 mm diameter, 40 mm long, Huatuo acupuncture needles, Suzhou, China) were vertically punctured at 15 ± 2 mm. After developing needle sensation, twirling at an angle of 90–180° and a frequency of 60–90 times/min and lifting and thrusting at a range of 0.3–0.5 cm and a frequency of 60–90 times/min were conducted. After manipulating the needle for 1 min, the needle was held in place for 30 min. During the 30 min, the physician repeated this manipulation for 1 min every 10 min.

#### 2.2.2. Resting-State fMRI Scan

The subjects were awake, with normal respiration, and laid supine on an examination bed. The head was placed in a foam headrest for maximum restriction of passive and active movements of the head. The subjects were instructed to avoid any systematic mental activity, and visual and audio stimulations were minimized with earplugs and eyeshades. Scanning was initiated once the subjects were familiarized with the circumstances.

Experiments were performed using a GE 3.0T MRI scanner with an 8-channel head coil. The MRI data (resting-state BOLD sequence) were collected at 15 min before needling and 15 min after withdrawing the needle. The scanning parameters are the same as those in our previous study [25]. The scanning methods are (1) transverse T1-weighted image (T1WI) sequence: 1 min, 51 s, fast spin echo sequence; OAx T1 FLAIR, repetition time: 1750 ms/echo time: 24 ms, inversion time: 960 ms, field of view: 24 cm × 24 cm/*Z*, matrix: 320 × 224/number of excitations = 1, thickness: 5.0 mm/interval: 1.0 mm, 30 slices total, echo train length: 8, and bandwidth: 31.25; (2) resting-state fMRI BOLD data collection: gradient echo-echo-planar imaging sequence scanning was conducted for 6 min in accordance with the following parameters: repetition time: 3000 ms/minimum, echo time: minimum, flip angle: 90, field of view: 240 mm × 240 mm, thickness: 5.0 mm/interval: 1.0 mm, 30 slices each time, and matrix: 96 × 96/number of excitations = 1.

#### 2.2.3. Image Processing and Analytical Methods

Preprocessing was performed using Data Processing Assistant for Resting-State fMRI (DPARSF V2.3; Yan & Zang, 2013, http://rfmri.org/DPARSF), which is based on Statistical Parametric Mapping (SPM8; members and collaborators of the Wellcome Trust Centre for Neuroimaging, 2009, http://www.fil.ion.ucl.ac.uk/spm/) and a Resting-State fMRI Data Analysis Toolkit (REST 1.8, Song et al., 2012, http://www.restfMRI.net/).

The preprocessing procedures are the same as those in our previous study [25], which include (1) converting DICOM to NIFTI, (2) slice timing after removing first 10 time points, (3) realigning and excluding subjects with max head motion > 1.5 mm on any axis and head rotation > 1.5 degrees, (4) segmenting and affixer regularization according to East Asians, (5) normalizing by using EPI templates, (6) smoothing images with a Gaussian kernel with isotropic full-width at half-maximum (FWHM) of 6 mm, and (7) removing the linear tendency of the data.

#### 2.2.4. Functional Connectivity Analysis

Generally, these methods can be classified into two categories: model-based methods and data-driven methods. Each category has its own merits and limitations; now many functional connectivity explorations are model-based. These studies select some regions of interest (ROIs) as so-called “seeds” and determine whether other regions are connected to these seeds by defining certain metrics and thereby generate the connectivity map of human brain [26].

As SO and Pa may be the key role in the CNS in cardiovascular regulation, we selected them as the seed point regions to explore modulated brain network changes underlying acupuncture hypertension treatment. We defined two ROIs according to Baroncini et al. [27]: the bilateral SO (*x*: ±6.1; *y*: 0.5; *z*: −15.0, radius 1 mm) and Pa (*x*: ±2.2; *y*: −1.4; *z*: −12.3, radius 1 mm). We defined SO (right: R) as ROI 1, SO (left: L) as ROI 2, Pa (R) as ROI 3, and Pa (L) as ROI 4.

#### 2.2.5. Statistical Analysis

Data were analyzed using REST 1.8 software. In the statistical analysis,* t*-test was used to explore the differences. Paired *t*-test was used in groups and two-sample* t*-test between groups. REST 1.8 software viewer was employed to identify the precise anatomical position in the brain with statistical significance on the corresponding MNI coordinate (AlphaSim correction *P* < 0.05; continuous voxel > 228). The results are presented as images visualized with the BrainNet Viewer (Xia et al., 2013; http://www.nitrc.org/projects/bnv/).

## 3. Results

### 3.1. Physiological Data

#### 3.1.1. The Change of SBP and DBP

There were no statistical differences observed between LR3 group and sham group in SBP and DBP before acupuncture, after once acupuncture, and after treatment. There was statistical difference in SBP in LR3 group after treatment compared to preacupuncture, *P* = 0.006, which could demonstrate that SBP decreased in LR3 group after treatment (Table 2).

#### 3.1.2. The Changes of SF-36 and QLICD-HY Scores

In the SF-36 survey for QoL, participants in the LR3 group reported the scores before acupuncture of physical functioning (PF, 85.71 ± 12.69), role physical (RP, 75.00 ± 36.69), bodily pain (BP, 79.07 ± 14.85), general health (GH, 50.64 ± 14.05), vitality (VI, 68.21 ± 18.57), social functioning (SF, 82.54 ± 15.54), role emotional (RE, 73.81 ± 35.03), and mental health (MH, 68.86 ± 14.98) and the scores after treatment of PF (84.29 ± 11.74), RP (76.78 ± 34.62), BP (80.14 ± 9.46), GH (52.50 ± 14.29), VI (67.86 ± 11.56), SF (89.68 ± 12.68), RE (76.19 ± 42.22), and MH (66.29 ± 15.51). Participants in the sham group reported the scores before acupuncture of PF (91.79 ± 5.04), RP (96.43 ± 9.08), BP (87.14 ± 9.14), GH (66.21 ± 11.73), VI (66.79 ± 10.12), SF (87.30 ± 16.22), RE (90.48 ± 27.51), and MH (75.43 ± 14.19) and the scores after treatment of PF (91.43 ± 9.89), RP (76.78 ± 34.62), BP (80.14 ± 9.46), GH (52.50 ± 14.29), VI (67.86 ± 11.56), SF (89.68 ± 12.68), RE (76.19 ± 42.22), and MH (66.29 ± 15.51). Participants in the sham group reported the scores before acupuncture of PF (91.79 ± 5.04), RP (91.07 ± 25.21), BP (84.68 ± 12.63), GH (69.00 ± 18.43), VI (72.86 ± 14.37), SF (89.68 ± 15.38), RE (92.86 ± 26.73), and MH (72.29 ± 17.08). There was no statistical difference observed between LR3 group and sham group in SF-36 before acupuncture and after treatment.

In the QLICD-HY, participants in the LR3 group reported the scores of physical domain (PHD, 69.65 ± 10.50), psychological domain (PSD, 79.55 ± 14.21), social domain (SOD, 78.90 ± 13.61), specific domain (SPD, 68.80 ± 14.69), and total (TOT, 73.82 ± 11.70) and the scores after treatment of PHD (67.41 ± 10.74), PSD (81.98 ± 7.64), SOD (77.43 ± 13.59), SPD (75.10 ± 11.44), and TOT (75.94 ± 8.70). Participants in the sham group reported the scores before acupuncture of PHD (73.22 ± 14.44), PSD (76.14 ± 16.84), SOD (65.42 ± 16.97), SPD (73.21 ± 12.60), and TOT (71.66 ± 13.08) and the scores after treatment of PHD (77.90 ± 13.11), PSD (86.36 ± 13.43), SOD (72.73 ± 17.47), SPD (73.31 ± 9.92), and TOT (79.18 ± 9.00). Participants in the LR3 group reported increased scores of SPD (*P* < 0.05). Changes in PHD, PSD, SOD, and TOT did not differ among participants.

### 3.2. Functional Connectivity

#### 3.2.1. The Changes in Functional Connectivity before and after Once Acupuncture in LR3 Group

**Figure 3:**
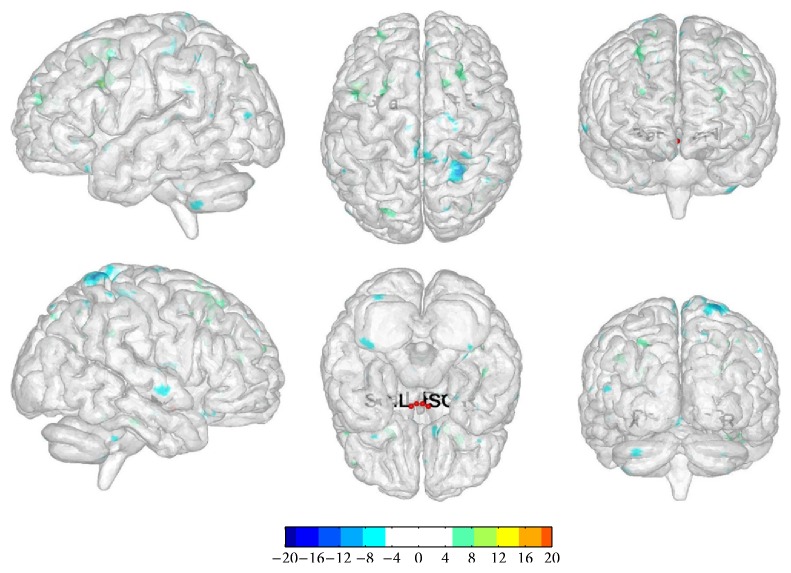
The changes in functional connectivity before and after once acupuncture in LR3 group. Note: the color bar represents intensity and red dots represent the seeds of SO and Pa. L is short for left and R is short for right. The single map represents change in functional connectivity obtained from combining results from all four seed points.

We selected bilateral SO and Pa as the seed point regions to explore functional connectivity changes before and after once acupuncture in LR3 group: (1) ROI 1: there were functional connectivity changes in right cerebrum superior frontal gyrus and left cerebrum sublobar extranuclear gyrus. (2) ROI 2: there were functional connectivity changes in right cerebrum frontal lobe subgyrus, left cerebrum frontal lobe subgyrus, and limbic lobe cingulate gyrus. (3) ROI 3: there were functional connectivity changes in right cerebrum frontal lobe medial frontal gyrus and left cerebrum frontal lobe medial frontal gyrus. (4) ROI 4: there were functional connectivity changes in right cerebrum frontal lobe middle frontal gyrus, medial frontal gyrus, and left cerebrum frontal lobe middle frontal gyrus (Table 3, Figure 3).

#### 3.2.2. The Changes in Functional Connectivity before and after Once Acupuncture in Sham Group

**Figure 4:**
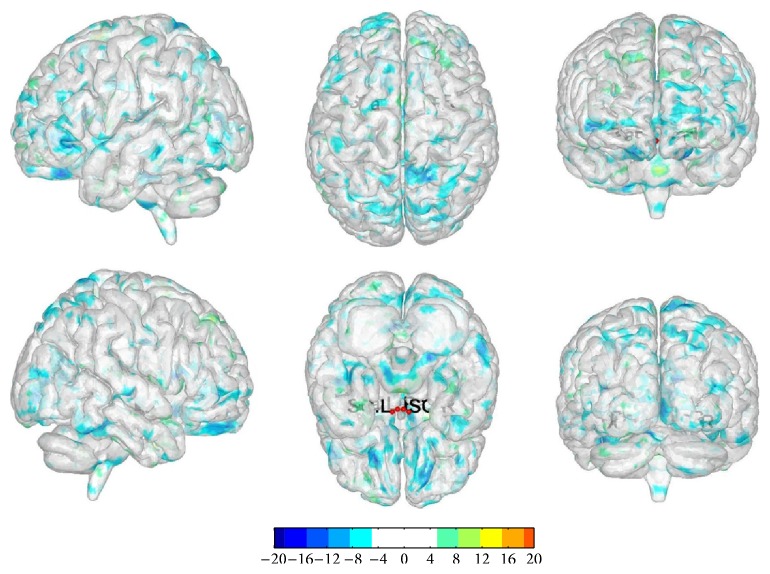
The changes in functional connectivity before and after once acupuncture in sham group. Note: the color bar represents intensity and red dots represent the seeds of SO and Pa. L is short for left and R is short for right. The single map represents change in functional connectivity obtained from combining results from all four seed points.

We selected bilateral SO and Pa as the seed point regions to explore functional connectivity changes before and after once acupuncture in sham group: (1) ROI 1: there were functional connectivity changes in right cerebrum frontal lobe subgyrus. (2) ROI 2: there were functional connectivity changes in left cerebrum limbic lobe anterior cingula. (3) ROI 3: there were functional connectivity changes in right cerebrum frontal lobe superior frontal gyrus, occipital lobe precuneus, and left cerebrum frontal lobe inferior frontal gyrus. (4) ROI 4: there were functional connectivity changes in left cerebrum limbic lobe anterior cingula (Table 4, Figure 4).

#### 3.2.3. The Changes in Functional Connectivity before and after Treatment Acupuncture in LR3 Group

**Figure 5:**
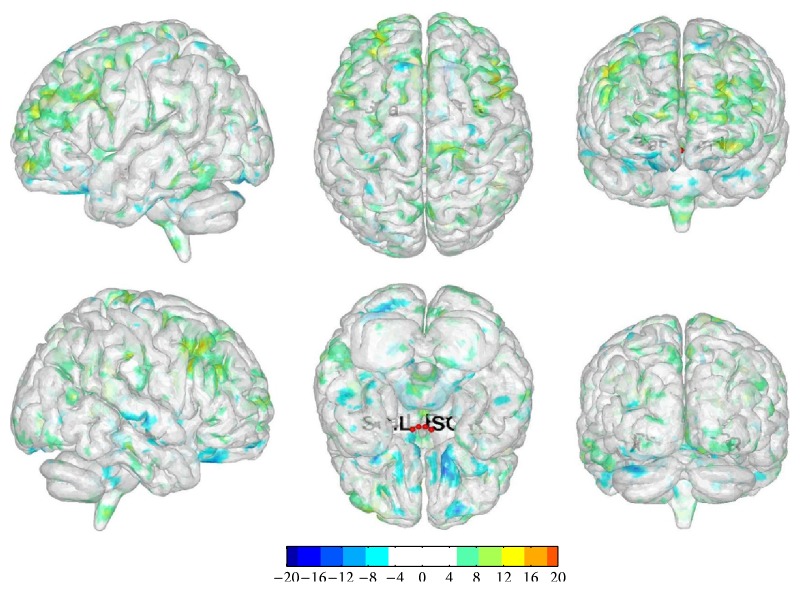
The changes in functional connectivity before and after treatment acupuncture in LR3 group. Note: the color bar represents intensity and red dots represent the seeds of SO and Pa. L is short for left and R is short for right. The single map represents change in functional connectivity obtained from combining results from all four seed points.

We selected bilateral SO and Pa as the seed point regions to explore functional connectivity changes before and after treatment acupuncture in LR3 group: (1) ROI 1: there were functional connectivity changes in right cerebrum frontal lobe subgyrus and left cerebrum limbic lobe medial frontal gyrus. (2) ROI 2: there were functional connectivity changes in right cerebrum frontal lobe superior frontal gyrus and inferior frontal gyrus. (3) ROI 3: there were functional connectivity changes in right cerebellum posterior lobe declive and left cerebrum limbic lobe cingulate gyrus. (4) ROI 4: there were functional connectivity changes in right cerebrum frontal lobe middle frontal gyrus, sublobar insula, and left cerebrum frontal lobe middle frontal gyrus (Table 5, Figure 5).

## 4. Discussion

The role of supraoptic (SO) nucleus and paraventricular (Pa) nucleus of the hypothalamus was secreting vasopressin (AVP), which could interact with neurons of cardiovascular activity, affecting cardiovascular activity, as well as influencing the body's baroreflex sensitivity [28]. Thus we selected SO and Pa as our seed points to explore functional connectivity changes in brain areas and demonstrate the mechanism of regulation in blood pressure.

In our study, there was only decrease in SBP after treatment compared to preacupuncture in LR3 group. However, some large scale hypertension studies have investigated and discussed the therapeutic effects of acupuncture treatment; in those studies Pandian et al. [6] randomized 160 hypertensive participants in a single-blind 6-week trial using 22 acupoints. Yin et al. [8] recruited 41 hypertensive or prehypertensive patients and designed a randomized double-blind 8-week trial using 14 acupoints. On the other side, there was no significant difference among individualized traditional Chinese acupuncture group (IND), standardized acupuncture group (STD), and invasive sham acupuncture group (CNTL) in SHARP [9] study. So we consider the assumption of acupuncture effect in hypertension which is long-term and numbers of acupoints.

There was no significant difference in SF-36 and QLICD-HY scores except for SPD in QLICD-HY. The SPD was the specific module in QLICD-HY questionnaires, which could reflect the symptom of headache, dizziness, palpitation, shortness, and anxiety of drugs. These symptoms are the most common and most directly affecting the quality of life in patients with hypertension. We could improve these problems by acupuncturing at LR3.

In our study, the changes of functional connectivity may be related to multiple components; one of them was acupuncture. The acupuncture effect could modulate the blood pressure, while the functional connectivity map could reflect the modulation in the brain network.

Compared with sham, acupuncture at LR3 instantaneous effects in the functional connectivity with seed points was more concentrated in the frontal lobe. Frontal lobe closely associated with emotions [29, 30] and emotions had relationship with blood pressure. So we guess frontal lobes could connect with the function of the hypothalamus and indirectly influence the regulation of blood pressure by acupuncture at LR3.

Compared with instantaneous effects, acupuncture at LR3 short-term effects in the functional connectivity with seed points had more regions in frontal lobe, cerebellum, and insula. However the limbic system could have functional connectivity with seed points in both LR3 and sham groups. Study [31] demonstrated that the limbic system had an important role in cardiovascular center. Besides, one research [32] showed stabbing pain could activate some parts of the anterior cingula. We could explain the reason why the limbic system could have connectivity in both groups. Although the role of cerebellum in cardiovascular center was unclear, there was an animal experiment that showed that the cerebellum had a relationship with nitric oxide synthase activity, indirectly related to blood pressure regulation. A case reported [33] resection cerebellar vermis and right cerebellum hemisphere in a cerebellum hemorrhage patient could cause transient orthostatic hypotension, indicating that the cerebellum was involved in blood pressure regulation. In our study, the connectivity between the cerebellum and seed points may reveal that the cerebellum has a role in blood pressure regulation.

We also believed that the improvement of quality of life in hypertensive patients was related to connectivity with frontal lobe and limbic system. Sabbatini et al. [34] suggested that microcapillary hemorrhage or inflammation may be associated with mild cognitive impairment in hypertensive rats, and the frontal lobe was closely related to cognitive function. Li et al. [35] considered the mechanisms of cognitive dysfunction in hypertensive patients may be because of abnormality in frontal lobe function connections. Onoda et al. [36] reported blood flow would be reduced in bilateral anterior cingulate gyrus and corpus callosum area in hypertensive patients by brain functional imaging. The decrease in anterior cingula and bilateral insula would further affect patients' cognitive abilities. So we suggested that acupuncture at LR3 could strengthen the connection between the frontal lobe, anterior cingula, insula, and hypothalamus, thus improving disease damage and compensating the function of cognition.

There are some limitations in our study: (1) Each group had not enough participants, and these participants did not stop taking antihypertensive drug or took the same one. (2) We did not use 24-hour ambulatory blood pressure monitoring to monitor the blood pressure. (3) The MRI we used did not involve the brain stem, which is one of the cardiovascular control centers. So we ignored the part of functional connectivity.

## 5. Conclusion

In our study, there was only decrease in SBP after treatment compared to preacupuncture in LR3 group. Compared with sham, acupuncture at LR3 instantaneous effects in the functional connectivity with seed points was more concentrated in the frontal lobe. And compared with instantaneous effects, acupuncture at LR3 short-term effects in the functional connectivity with seed points had more regions in frontal lobe, cerebellum, and insula. These regions may be closely related to hypertension.

## Figures and Tables

**Table 1:** Baseline characteristics ($\overset{-}{X}$ ± *S*).

Characteristic	LR3 group	Sham group	*P*
Gender			
Male/female	2/13	6/9	0.22
Age (year)	56.53 ± 7.52	56.73 ± 4.91	0.57
Heredity			
Yes/no	8/7	11/4	0.37
Course of disease (month)	106.00 ± 146.05	84.53 ± 62.52	0.49
Medication			
Yes/no	8/7	9/6	0.78

**Table 2:** Physiological data of LR3 and sham groups ($\overset{-}{X}$ ± *S*).

	SBP (mmHg)	DBP (mmHg)
LR3 group		
B	134.93 ± 9.05	86.29 ± 7.35
A	134.14 ± 9.94	86.00 ± 8.56
T	129.71 ± 8.66^*∗*^	86.71 ± 6.11

Sham group		
B	136.86 ± 18.26	87.86 ± 11.22
A	134.21 ± 18.26	86.93 ± 8.19
T	132.64 ± 12.97	84.21 ± 7.47

B: before acupuncture, A: after once acupuncture, and T: after treatment. The SBP in LR3 group after treatment compared to before acupuncture, ^*∗*^ *P* = 0.006.

**Table 3:** The changes in functional connectivity before and after once acupuncture in LR3 group.

ROI	Regions	HS	BA	Cluster size (voxels)	Peak intensity	Peak MNI coordinate		
ROI 1	Superior frontal gyrus	R	10	581	−5.5292	21	51	24
	Sublobar extranuclear	L	—	512	5.7772	−9	−18	24

ROI 2	Frontal lobe subgyral	R	—	233	5.1657	27	24	39
	Frontal lobe subgyral	L	—	332	4.6882	−15	24	45
	Limbic lobe cingulate gyrus	L	24	337	−5.2389	−6	−12	42

ROI 3	Frontal lobe medial frontal gyrus	R	—	287	5.4445	9	42	21
	Frontal lobe medial frontal gyrus	L	—	260	−5.64	−9	−15	51

ROI 4	Frontal lobe middle frontal gyrus	R	—	319	5.253	36	12	42
	Frontal lobe medial frontal gyrus	R	—	256	4.5799	9	45	21
	Frontal lobe middle frontal gyrus	L	9	284	6.6073	−42	15	33

HS: hemisphere, BA: Brodmann's area.

**Table 4:** The changes in functional connectivity before and after once acupuncture in sham group.

ROI	Regions	HS	BA	Cluster size (voxels)	Peak intensity	Peak MNI coordinate		
ROI 1	Frontal lobe subgyral	R	—	493	−5.4482	18	33	−6

ROI 2	Limbic lobe anterior cingulate	L	—	325	−5.5452	−6	33	−3

ROI 3	Frontal lobe superior frontal gyrus	R	—	235	−5.256	18	54	−21
	Occipital lobe precuneus	R	—	308	−5.9429	15	−66	18
	Frontal lobe inferior frontal gyrus	L	—	244	−5.1067	−18	36	−24

ROI 4	Limbic lobe anterior cingulate	L	—	245	−4.683	−9	30	−6

HS: hemisphere, BA: Brodmann's area.

**Table 5:** The changes in functional connectivity before and after treatment acupuncture in LR3 group.

ROI	Regions	HS	BA	Cluster size (voxels)	Peak intensity	Peak MNI coordinate		
ROI 1	Frontal lobe subgyral	R	—	851	6.1613	36	−15	39
	Limbic lobe medial frontal gyrus	L	—	795	8.8247	−6	36	30

ROI 2	Frontal lobe superior frontal gyrus	R	—	365	8.7245	6	9	60
	Frontal lobe inferior frontal gyrus	R	—	316	5.4087	51	12	24

ROI 3	Posterior lobe declive	R		268	5.045	24	−78	−21
	Limbic lobe cingulate gyrus	L	—	1895	6.5397	−3	27	36

ROI 4	Frontal lobe middle frontal gyrus	R		413	6.8551	36	12	45
	Sublobar insula	R	13	510	5.2198	45	−42	18
	Frontal lobe middle frontal gyrus	L		880	5.0709	−45	6	51

HS: hemisphere, BA: Brodmann's area.
